# ATR inhibition enhances radiosensitivity in p53-deficient colorectal cancer cells

**DOI:** 10.1007/s12672-026-05692-9

**Published:** 2026-07-30

**Authors:** Na Shen, Rui Zong, Jie Li, Xuemei Liu

**Affiliations:** 1 Department of Oncology, Suining Central Hospital, China, No. 27, Dongping North Road, Hedong New District, Chuanshan District, Suining, 629000 China; 2 First Department of Oncology Radiotherapy, Qingdao Central Hospital, University of Health and Rehabilitation Sciences (Qingdao Central Hospital), Qingdao, 266042 China; 3 Department of Oncology, School of Medicine, Mianyang Central Hospital, University of Electronic Science and Technology, Mianyang, 621000 China

**Keywords:** Colorectal cancer, Ataxia telangiectasia and Rad3-related (ATR), p53, Radiation

## Abstract

Radiosensitizers that improve tumor-cell responses to radiation without increasing normal-tissue toxicity are needed in colorectal cancer (CRC). We investigated whether inhibition of ataxia telangiectasia and Rad3-related (ATR) kinase by VE-822 (berzosertib) enhances radiation response and whether p53 status is associated with the magnitude of response. HCT116 (p53 wild type), HT29 (p53 mutant), and HCT116 cells with siRNA-mediated p53 knockdown (HCT116 KD) were treated with VE-822 with or without ionizing radiation (RT). Short-term cell viability and long-term clonogenic survival were evaluated. DNA-damage signaling and ATR-pathway activity were assessed by analysis of γH2AX and phosphorylated CHK1, whereas flow cytometry was used to examine cell-cycle distribution, mitotic entry, and apoptosis. VE-822 IC50 values were 80.8 nM for HCT116, 33.6 nM for HT29, and 19.6 nM for HCT116 KD cells. A low concentration of VE-822 (20 nM, administered 1 h before RT) had minimal effects in HCT116 cells but reduced viability and clonogenic survival in HCT116 KD cells, particularly when combined with RT. VE-822 reduced pCHK1 and was associated with sustained γH2AX signaling, with more pronounced effects in HCT116 KD cells. At 24 h after RT, HCT116 cells predominantly accumulated in G1 with modest apoptosis, whereas HCT116 KD cells showed greater G2/M accumulation and apoptosis after VE-822 plus RT. Increased phospho-Histone H3 positivity after combined treatment was consistent with increased mitotic entry. These in vitro findings suggest that impaired p53 function is associated with increased sensitivity to combined ATR inhibition and irradiation and support further evaluation of p53 status as a candidate response biomarker.

**Supplementary Information:**

The online version contains supplementary material available at https://doi.org/10.1007/s12672-026-05692-9.

## Introduction

Colorectal cancer (CRC) remains one of the most prevalent malignancies worldwide and a leading cause of cancer-related death [1, 2]. Despite incremental gains from modern chemotherapy, targeted agents, and immunotherapy, outcomes for patients with locally advanced or metastatic disease remain suboptimal [3]. Radiotherapy (RT) is used across clinical scenarios, from neoadjuvant treatment of rectal cancer to palliation of metastatic lesions, but the therapeutic ratio is constrained by normal-tissue tolerance, underscoring the need for tumor-selective radiosensitizers that do not exacerbate toxicity [4–6].

TP53 is among the most frequently altered genes in CRC [7, 8]. The p53 tumor suppressor safeguards genome integrity by integrating DNA-damage signals to induce cell-cycle arrest, DNA repair, senescence, or apoptosis. Loss-of-function mutations abrogate canonical p53 activity, while certain gain-of-function variants promote invasion, metastasis, and therapy resistance [7]. Functionally, p53 loss compromises the G1 checkpoint, forcing tumor cells to rely disproportionately on S/G2 checkpoints to survive endogenous and therapy-induced replication stress—an exploitable vulnerability for S/G2 checkpoint–targeted agents [9].

Ataxia telangiectasia and Rad3-related (ATR) kinase, together with its downstream effector checkpoint kinase 1 (CHK1), is a master regulator of replication-stress tolerance [10]. Activated by RPA-coated single-stranded DNA generated at stalled replication forks, ATR signaling preserves replication fork integrity by activating CHK1, limiting unscheduled origin firing, preventing fork collapse, and promoting DNA repair and fork restart. ATR also coordinates homologous recombination and enforces the G2/M checkpoint, thereby preventing mitotic entry in the presence of unrepaired DNA damage [11]. Pharmacologic ATR inhibition (ATRi) disables these safeguards and can convert sublethal replication stress into lethal damage, particularly when combined with DNA-damaging modalities such as ionizing radiation [12]. Several clinically advanced ATR inhibitors (e.g., berzosertib/VE-822, ceralasertib, elimusertib, camonsertib) have shown manageable safety and radiosensitization in preclinical models, and early clinical studies support the feasibility of ATRi–RT combinations [13–15]. Mechanistically, ATR inhibition suppresses CHK1 phosphorylation, impairs homologous recombination repair, and prolongs radiation-induced DNA damage [16]. These effects are particularly relevant in p53-deficient tumours, which are more dependent on the ATR–CHK1 pathway for cell-cycle checkpoint control. Consequently, ATR inhibition may preferentially radiosensitize p53-deficient cancer cells and improve treatment response.

We hypothesized that ATR inhibition enhances the cellular response to radiotherapy in colorectal cancer and that the magnitude of this effect is influenced by p53 status. Using colorectal cancer models with wild-type, mutant, and siRNA-reduced p53 expression, we evaluated the effects of ATR inhibition on radiation response and explored cellular mechanisms associated with treatment sensitivity.

## Materials and methods

### Cell culture

Human CRC cell lines HCT116 (p53 WT) and HT29 (p53 mutant) were obtained from the Shanghai Cell Bank (China) and authenticated by STR profiling. Cells were cultured in DMEM (Millipore Sigma) supplemented with 10% FBS and 1% penicillin/streptomycin at 37 °C, 5% CO₂. Mycoplasma testing was performed routinely.

### siRNA-mediated p53 knockdown

HCT116 cells were seeded in 6-well plates (2 × 10⁵ cells/well) to reach approximately 40–50% confluence at the time of transfection. TP53-targeting siRNA (siP53) and a non-targeting control siRNA (siNC) were synthesized by GenePharma (Shanghai, China). Cells were transfected using Lipofectamine™ RNAiMAX Transfection Reagent (Thermo Fisher Scientific, Waltham, MA, USA) in Opti-MEM™ Reduced Serum Medium (Thermo Fisher Scientific, Waltham, MA, USA) at a final siRNA concentration of 20 nM, following manufacturer instructions. After a 15-min incubation at room temperature to allow complex formation, the mixture was added dropwise to the cells in antibiotic-free culture medium. Following 6 h of incubation, the medium was replaced with complete growth medium containing 10% fetal bovine serum. Cells were harvested at 72 h post-transfection for subsequent assays. Western blotting was performed using an anti-p53 antibody (Cell Signaling Technology, Danvers, MA, USA; rabbit monoclonal; 1:1000 dilution) and an anti-GAPDH antibody (Cell Signaling Technology, Danvers, MA, USA; rabbit monoclonal; 1:5000 dilution). Band intensities were quantified using ImageJ software (National Institutes of Health, Bethesda, MD, USA). p53 expression was normalized to GAPDH and expressed relative to the siNC control group. Knockdown efficiency was calculated as: KD efficiency (%) = [1 − (normalized p53 expression in siP53 / normalized p53 expression in siNC)] × 100.

### Cell viability assay (MTT assay)

Human colorectal cancer HCT116, HT29, and HCT116 p53-knockdown (HCT116 KD) cells were seeded in 96-well plates at a density of 2 × 10^5 cells/well. After overnight attachment, cells were treated with increasing concentrations of VE-822 (berzosertib; VX-970; Selleck Chemicals, Houston, TX, USA) diluted in culture medium; control wells received vehicle alone. For dose-response experiments, cells were exposed to VE-822 for 48 h. For combination experiments, cells received vehicle or VE-822 (20 nM) 1 h before irradiation with 4 Gy, and viability was assessed 48 h after irradiation. MTT solution (20 µl; 3-(4,5-dimethylthiazol-2-yl)-2,5-diphenyltetrazolium bromide; Sigma-Aldrich, St. Louis, MO, USA) was added to each well and incubated for 4 h at 37 °C. The medium was then aspirated, the formazan crystals were dissolved in 150 µl dimethyl sulfoxide, and absorbance was measured at 570 nm using an Infinite M Plex multifunctional microplate reader (Tecan Group Ltd., Männedorf, Switzerland). Cell viability was calculated relative to the vehicle-treated control group.

### Clonogenic survival assay

HCT116, HT29, and HCT116 KD cells were seeded in 6-cm dishes at 200 cells per dish. Cells were assigned to vehicle control, VE-822 alone (20 nM), irradiation alone (4 Gy), or VE-822 plus irradiation. VE-822 was added 1 h before irradiation. Irradiation was delivered using an X-RAD 320i X-ray machine (Precision X-Ray, Inc., USA) at a dose rate of 1.0 Gy/min. Culture medium was replaced every 3–4 days. After 14 days, colonies were fixed with methanol, stained with Giemsa, and colonies containing at least 50 cells were counted. Clonogenic survival was expressed relative to the corresponding untreated control.

### Flow-cytometric analysis of cell cycle distribution, apoptosis and phospho-Histone H3-positive cells

For cell-cycle analysis, cells were seeded in six-well plates and allowed to reach approximately 70–80% confluence. Cells were assigned to vehicle control, VE-822 (20 nM), irradiation (4 Gy), or VE-822 plus irradiation. VE-822 was added 1 h before irradiation. Cells were harvested 24 h after irradiation by trypsinization, washed twice with phosphate-buffered saline (PBS; Gibco, Thermo Fisher Scientific, Waltham, MA, USA), and fixed in 70% ethanol at 4 °C for 2 h. Fixed cells were washed with PBS, incubated with RNase A (50 µg/ml; Sigma-Aldrich, St. Louis, MO, USA) at 37 °C for 30 min, and stained with propidium iodide (PI; 50 µg/ml; Sigma-Aldrich) for 30 min in the dark. Cell-cycle distribution was analyzed using a BD FACSCalibur flow cytometer (BD Biosciences, San Jose, CA, USA), and data were processed using ModFit software (Verity Software House).

To assess mitotic entry following ATR inhibition and irradiation, HCT116 and HCT116 KD cells were synchronized using a double thymidine block. Cells were treated with 2 mM thymidine (Sigma-Aldrich) for 16 h, released into fresh medium for 8 h, and then subjected to a second 16-h thymidine block. Following release from the second block, cell-cycle progression was assessed at serial time points. Based on preliminary synchronization optimization, the 7-h time point after release was selected for the subsequent treatment experiments; representative cell-cycle profiles at this time point are provided in Supplementary Figure S4. At 7 h after release, synchronized cells were treated with vehicle control, VE-822 (20 nM), irradiation, or VE-822 plus irradiation. VE-822 was added 1 h before irradiation and maintained throughout the experiment. Cells were harvested 9 h after irradiation, fixed in ice-cold 70% ethanol overnight at 4 °C, washed twice with PBS, and resuspended in blocking buffer consisting of PBS supplemented with 2% bovine serum albumin, 0.1% Tween-20, and 0.1% Triton X-100. After blocking for 1 h at room temperature, cells were incubated with anti-phospho-Histone H3 (Ser10) primary antibody (1:200; Cell Signaling Technology, Danvers, MA, USA) for 2 h at room temperature. After three washes, cells were incubated with Alexa Fluor 488-conjugated secondary antibody (1:500; Thermo Fisher Scientific) for 1 h at room temperature in the dark. Samples were analyzed using a BD FACSCalibur flow cytometer, and data were processed using FlowJo software (Tree Star Inc., Ashland, OR, USA). Mitotic entry was quantified as the percentage of pH3-positive cells in the total cell population. Apoptosis was assessed 24 h after treatment. Both floating and adherent cells were collected, washed twice with PBS, and stained using an Annexin V/propidium iodide-based apoptosis assay according to the manufacturer’s instructions. Samples were analyzed using a BD FACSCalibur flow cytometer, and the percentages of early and late apoptotic cells were quantified using FlowJo software.

### Western blotting

Western blot analysis was performed 24 h after treatment. Cells were lysed in RIPA buffer (Thermo Fisher Scientific, Waltham, MA, USA) supplemented with protease inhibitor cocktail (Roche Diagnostics, Basel, Switzerland). Protein concentrations were determined using the Bio-Rad Protein Assay Kit (Bio-Rad Laboratories, Hercules, CA, USA). Equal amounts of protein were separated by SDS-PAGE and transferred onto polyvinylidene difluoride membranes (MilliporeSigma, Burlington, MA, USA). Membranes were blocked with 5% non-fat milk in TBST and incubated overnight at 4 °C with primary antibodies against CHK1, phospho-CHK1 (Ser345), γH2AX (Ser139), phospho-Histone H3 (Ser10), cleaved caspase-3, p53, and GAPDH (Cell Signaling Technology, Danvers, MA, USA; 1:1000 unless otherwise specified). After washing with TBST, membranes were incubated with horseradish peroxidase-conjugated secondary antibodies (Cell Signaling Technology; 1:5000) for 1 h at room temperature. Band intensities were quantified using ImageJ. Total protein expression was normalized to GAPDH, and phosphorylated proteins were normalized to their corresponding total protein when available. Original, unprocessed, as-full-length-as-available blot images are provided in Supplementary Figures S1–S3; red boxes identify the regions used in the main figures.

### Statistical analysis

Statistical analyses were performed using SPSS software (version 14.0; IBM Corp., Armonk, NY, USA). Data are presented as mean ± standard deviation (SD), unless otherwise specified. Differences between groups were evaluated using one-way analysis of variance (ANOVA), and a p-value < 0.05 was considered statistically significant. Graphs were generated using GraphPad Prism version 9 (GraphPad Software, San Diego, CA, USA). For cell viability assays, half-maximal inhibitory concentration (IC50) values were calculated by nonlinear regression using a four-parameter dose–response curve fit in GraphPad Prism. Western blot band intensities were quantified using ImageJ software (National Institutes of Health, Bethesda, MD, USA). Protein expression levels were normalized to GAPDH, while phosphorylated proteins were normalized to their corresponding total protein levels and expressed relative to the control group.

## Results

### ATR inhibition preferentially enhances radiation response in colorectal cancer cells with impaired p53 function

**Fig. 1:**
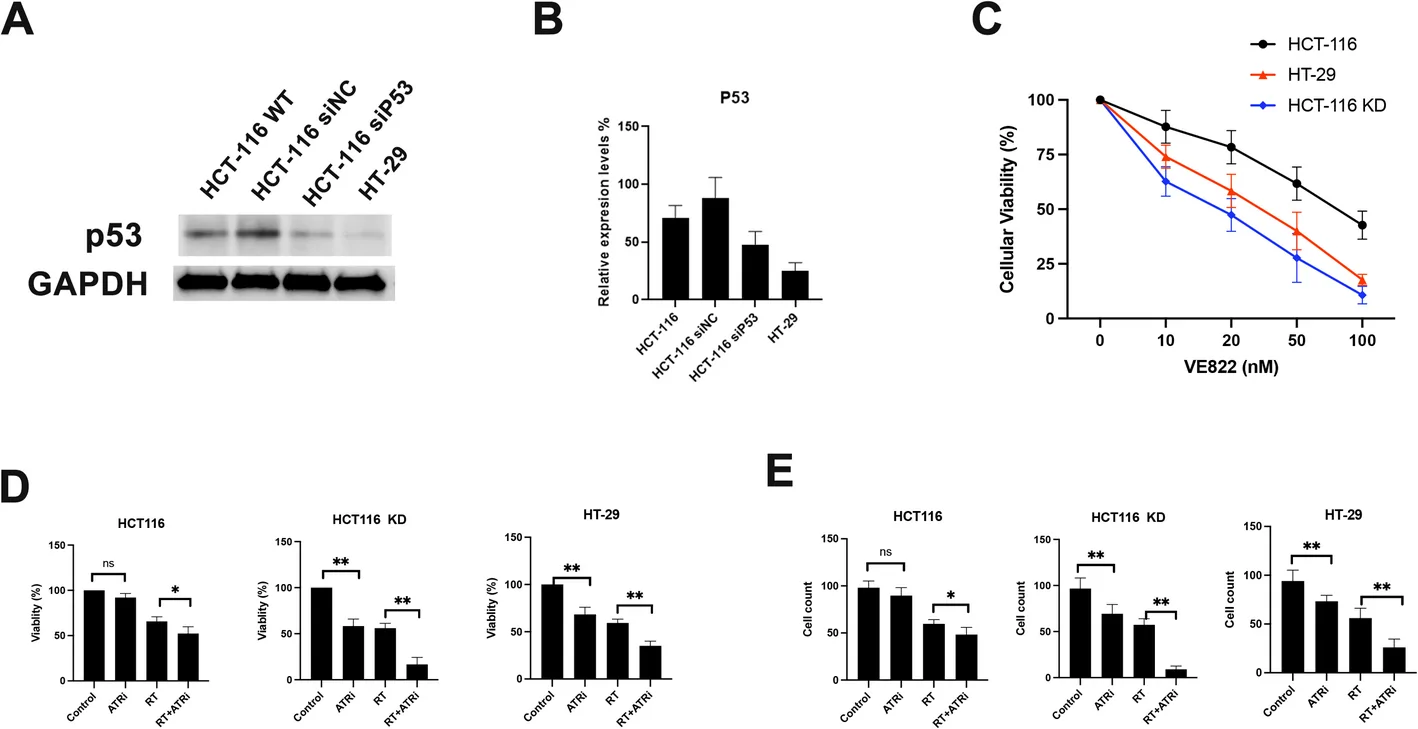
ATR inhibition preferentially enhances radiation response in colorectal cancer cells with impaired p53 function. **A **Western blot analysis of p53 expression in HCT116 siNC and HCT116 siP53 cells; GAPDH served as a loading control. **B **Quantification of p53 expression normalized to GAPDH. **C **VE-822 dose-response curves and IC50 values for HCT116, HT29, and HCT116 KD cells. **D **MTT viability 48 h after vehicle or VE-822 (20 nM, 1 h before irradiation) with or without 4 Gy irradiation. **E **Clonogenic survival after VE-822 with or without irradiation. Data are presented as mean ± SD from three independent experiments. Statistical significance was determined using one-way ANOVA. ns, not significant; *p < 0.05; **p < 0.01

To determine whether p53 status influences sensitivity to ATR inhibition, we evaluated the response to VE-822 in CRC models with distinct p53 backgrounds: HCT116 (p53 wild type), HT29 (p53 mutant), and HCT116 p53-knockdown (HCT116 KD) cells (Fig. 1A and Supplementary Figure S1). Western blot quantification demonstrated an approximately 53% reduction in p53 protein expression in siP53-transfected cells relative to the siNC control group, confirming partial p53 knockdown (Fig. 1B). MTT assays yielded IC50 values of 80.8 nM for HCT116, 33.6 nM for HT29, and 19.6 nM for HCT116 KD cells (Fig. 1C), indicating greater sensitivity to ATR inhibition in the p53-impaired models. To enable comparisons under minimally cytotoxic conditions, approximately one-quarter of the IC50 was initially used for each cell line (20 nM for HCT116, 10 nM for HT29, and 5 nM for HCT116 KD). A uniform VE-822 concentration of 20 nM was used for subsequent mechanistic and irradiation-combination experiments.

Short-term viability was assessed by MTT assay 48 h after irradiation (Fig. 1D). ATR inhibition had limited effects on HCT116 viability, either alone or combined with irradiation. In contrast, HT29 and HCT116 KD cells showed reduced viability after ATR inhibition, with a further reduction after combined treatment. Long-term clonogenic survival was then evaluated (Fig. 1E). Consistent with the viability data, HT29 cells showed greater sensitivity to ATR inhibition, particularly in combination with irradiation. HCT116 KD cells showed a similar response pattern, with reduced clonogenic survival after combined ATR inhibition and irradiation. Together, these findings suggest that impaired p53 function is associated with increased sensitivity to ATR inhibitor-mediated enhancement of radiation response in the tested CRC cell models.

### ATR inhibition suppresses ATR–CHK1 signaling and is associated with increased radiation-induced DNA-damage signaling

**Fig. 2:**
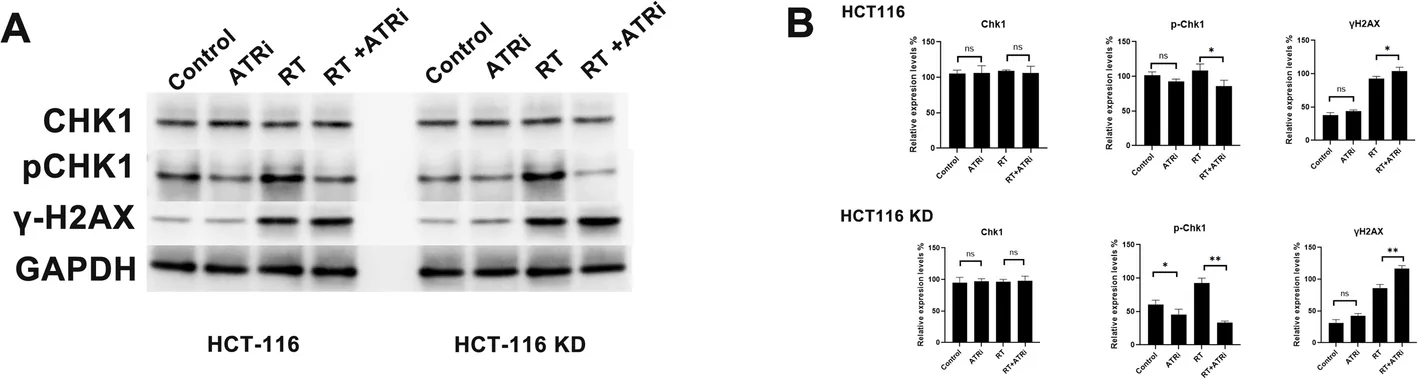
ATR inhibition suppresses ATR–CHK1 signaling and is associated with sustained radiation-induced DNA-damage signaling. **A** Western blot analysis of total CHK1, phosphorylated CHK1 (p-CHK1), and γH2AX in HCT116 and HCT116 KD cells after vehicle control, VE-822, irradiation, or combined treatment. **B** Densitometric quantification. Total CHK1 and γH2AX were normalized to GAPDH, and p-CHK1 was normalized to total CHK1. Data are presented as mean ± SD from three independent experiments. Statistical significance was determined using one-way ANOVA. ns, not significant; *p < 0.05; **p < 0.01

To investigate mechanisms associated with ATR inhibitor-mediated enhancement of radiation response, we examined ATR–CHK1 signaling and DNA-damage responses in HCT116 and HCT116 KD cells. Total CHK1 levels remained largely unchanged across treatment conditions (Fig. 2A, B and Supplementary Figure S2A). In HCT116 cells, ATR inhibition produced a modest reduction in phosphorylated CHK1, whereas the combination of ATR inhibition and irradiation reduced pCHK1 relative to irradiation alone (Fig. 2A, B and Supplementary Figure S2B). In HCT116 KD cells, pCHK1 suppression was more pronounced, with the lowest level observed after combined treatment. We next assessed γH2AX, a marker of DNA-damage signaling. γH2AX increased after irradiation in both cell lines and remained elevated when ATR inhibition was combined with irradiation (Fig. 2A, B and Supplementary Figure S2C, D). These observations indicate that VE-822 inhibits ATR–CHK1 checkpoint signaling and is associated with sustained γH2AX signaling at the 24-h time point, with more pronounced effects in cells with reduced p53 expression.

### ATR inhibition increases G2/M accumulation and mitotic entry after irradiation in cells with reduced p53 expression

**Fig. 3:**
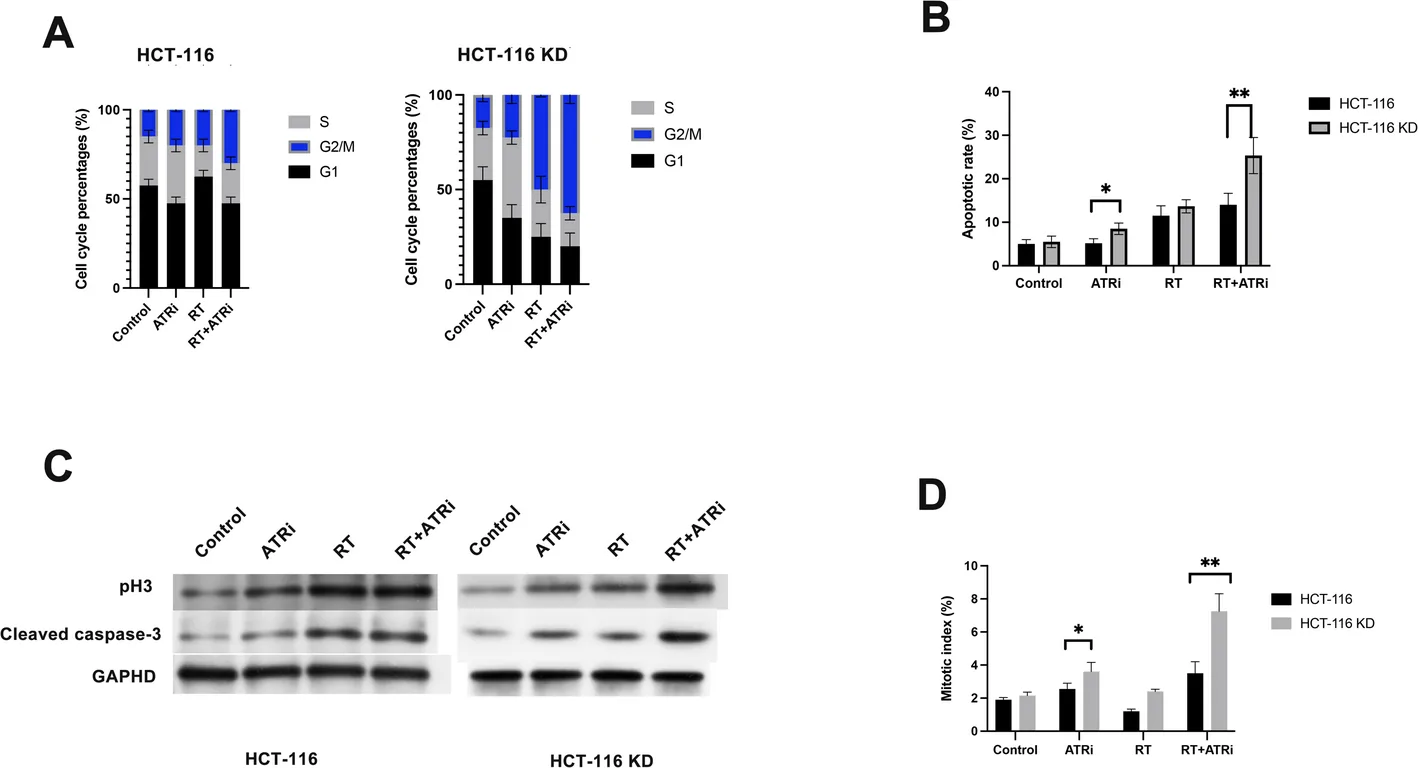
ATR inhibition increases G2/M accumulation, mitotic entry, and apoptosis after irradiation in cells with reduced p53 expression. **A** Cell-cycle distribution of HCT116 and HCT116 KD cells 24 h after treatment. **B **Quantification of apoptotic cells. **C** Western blot analysis of phospho-Histone H3 (pH3) and cleaved caspase-3. **D **Densitometric quantification of pH3 and cleaved caspase-3 normalized to GAPDH. **E **Flow-cytometric quantification of pH3-positive cells after synchronization and treatment. Data are presented as mean ± SD from three independent experiments. Statistical significance was determined using one-way ANOVA. *ns *not significant; *p < 0.05; **p < 0.01

To investigate how ATR inhibition influences cell-cycle progression after irradiation, we analyzed cell-cycle distribution 24 h after treatment. In HCT116 cells, irradiation predominantly induced G1 accumulation, consistent with an intact p53-dependent checkpoint, whereas ATR inhibition alone caused only modest changes. Combined ATR inhibition and irradiation partially reduced G1 accumulation and increased the G2/M fraction (Fig. 3A). In HCT116 KD cells, irradiation induced a greater G2/M accumulation, and the combined treatment further increased the G2/M fraction. Because a G2/M DNA-content profile does not distinguish G2 arrest from mitotic entry, we assessed phospho-Histone H3 (pH3). Western blotting showed increased pH3 after combined treatment, particularly in HCT116 KD cells (Fig. 3C, D and Supplementary Figure S3A), consistent with increased mitotic entry. For flow-cytometric assessment of mitotic entry, synchronized cells were treated at the selected 7-h time point after release from the second thymidine block (Supplementary Figure S4). In HCT116 cells, pH3-positive cells accounted for 2.0 ± 0.4% in the control group, 2.8 ± 0.5% after ATR inhibition, 1.2 ± 0.3% after irradiation, and 4.0 ± 0.6% after combined treatment (Fig. 3E). In HCT116 KD cells, the corresponding percentages were 2.3 ± 0.5%, 4.0 ± 0.6%, 2.5 ± 0.5%, and 9.0 ± 1.0%, respectively. The increase after combined treatment was greater in HCT116 KD cells than in HCT116 cells. These findings support the interpretation that ATR inhibition promotes mitotic entry despite irradiation-associated DNA damage, particularly when p53 expression is reduced.

### ATR inhibition enhances apoptosis after irradiation in cells with reduced p53 expression

We next assessed whether these cell-cycle changes were accompanied by increased cell death. Flow cytometry showed that ATR inhibition increased apoptosis in HCT116 KD cells relative to HCT116 cells after ATR inhibitor treatment alone (*p* < 0.05; Fig. 3B). Combined ATR inhibition and irradiation produced the highest apoptotic fraction in HCT116 KD cells (*p* < 0.01 versus RT). Consistent with the flow-cytometry findings, cleaved caspase-3 increased after ATR inhibition, with the strongest signal after combined treatment in HCT116 KD cells (Fig. 3C, D and Supplementary Figure S3B, C). These data indicate that reduced p53 expression is associated with greater apoptosis after combined ATR inhibition and irradiation. Together with the pH3 findings, the results are consistent with disruption of compensatory checkpoint control and inappropriate mitotic entry contributing to treatment-associated cell death.

## Discussion

The increasing resistance of colorectal cancer (CRC) to conventional chemotherapy, radiotherapy, and their combination represents a persistent clinical challenge [17–19]. One major mechanism underlying this resistance is the activation of DNA damage response and repair pathways that enable tumor cells to survive genotoxic stress. Understanding these adaptive mechanisms is essential for identifying new vulnerabilities and developing rational combination strategies that overcome resistance [20–22]. In this context, agents that disrupt cell-cycle checkpoints or DNA repair signaling can enhance the efficacy of radiotherapy (RT) by preventing efficient damage recovery.

In the present study, pharmacologic ATR inhibition enhanced the cellular response to ionizing radiation in the tested CRC models in vitro. The magnitude of this effect was associated with p53 functional status. VE-822 had limited single-agent effects in HCT116 cells with wild-type p53 but reduced viability in HT29 cells with mutant p53 and HCT116 cells with partial p53 knockdown. Combined ATR inhibition and irradiation produced greater reductions in viability and clonogenic survival in the p53-impaired models than in parental HCT116 cells, indicating a potentially exploitable dependence on ATR signaling.

Mechanistically, ATR inhibition reduced CHK1 phosphorylation and was associated with sustained γH2AX signaling after irradiation, consistent with impaired checkpoint signaling and prolonged DNA-damage signaling [23–25]. Flow cytometry revealed different checkpoint responses according to p53 status. In p53-wild-type HCT116 cells, irradiation predominantly induced G1 accumulation, whereas cells with reduced p53 expression relied more heavily on the G2/M checkpoint. Combined ATR inhibition and irradiation increased G2/M accumulation, pH3-positive mitotic entry, and apoptosis in HCT116 KD cells. These findings are consistent with the concept that reduced p53 function increases dependence on ATR–CHK1 signaling during replication and radiation-induced stress. However, increased pH3 and γH2AX are indirect markers; direct measurement of mitotic catastrophe and DNA-repair kinetics was not performed.

Our findings are consistent with ATR functioning as a central regulator of replication-stress tolerance and support further evaluation of ATR inhibition as a radiosensitizing strategy. The use of parental HCT116 and HCT116 p53-knockdown cells reduced confounding from differences in genetic background and supports a contribution of reduced p53 expression to the observed response. Nevertheless, the knockdown was transient and partial, and therefore does not establish a definitive causal relationship equivalent to a stable TP53 knockout model. The similar response pattern observed in HT29 cells suggests potential relevance to p53-mutant CRC, but HT29 and HCT116 cells differ in multiple molecular characteristics beyond TP53 status. Radiotherapy for locally advanced rectal cancer is commonly delivered with fluoropyrimidine-based chemotherapy such as 5-fluorouracil or capecitabine [26–28]. Future work should therefore evaluate ATR inhibitors with clinically relevant chemoradiotherapy regimens. The present data also provide a rationale for evaluating low-dose ATR inhibitor administration shortly before irradiation; however, normal-cell effects, normal-tissue toxicity, and the therapeutic window were not assessed and require dedicated preclinical evaluation.

Several limitations should be acknowledged. Most mechanistic analyses used a single radiation dose and a single post-treatment time point, limiting assessment of the temporal dynamics of ATR signaling, DNA-damage responses, cell-cycle redistribution, and apoptosis. Radiosensitization was evaluated primarily at 4 Gy rather than with complete radiation survival curves, and formal sensitizer enhancement ratios were not calculated. p53 suppression was achieved by transient siRNA-mediated knockdown and was partial, which may not be maintained throughout longer-term clonogenic assays and may not reproduce the biology of stable TP53 loss [29]. The IC50 values should be interpreted as approximate estimates because the dose-response curves were generated over a limited concentration range. The study included a small number of CRC models, and HT29 differs from HCT116 in multiple genetic characteristics beyond TP53 status [29–30]. No normal colorectal epithelial model was included, so tumor selectivity and the therapeutic window could not be directly assessed. In addition, γH2AX and pH3 provide indirect evidence of sustained DNA-damage signaling and mitotic entry, respectively; direct DNA-repair kinetics, chromosome abnormalities, and mitotic catastrophe were not measured. Finally, in vivo studies are required to assess efficacy, pharmacologic exposure, normal-tissue toxicity, and translational relevance.

In summary, ATR inhibition was associated with enhanced radiation response, reduced CHK1 phosphorylation, sustained γH2AX signaling, increased pH3-positive mitotic entry, and increased apoptosis in CRC cell models with impaired p53 function. These in vitro findings provide a rationale for further investigation of p53 status as a candidate biomarker for ATR inhibitor–radiotherapy combinations. Validation in stable genetic models, additional CRC backgrounds, normal-cell systems, and in vivo studies will be required before clinical conclusions can be drawn.

## Supplementary Information

Below is the link to the electronic supplementary material.


Supplementary Material 1.


## Data Availability

The datasets generated and/or analyzed during the current study are not publicly available due to the fact that there are still some related experiments in progress in our group but are available from the corresponding author on reasonable request.
