# Supplementary material for: ATR inhibition enhances radiosensitivity in p53-deficient colorectal cancer cells
Source: Discov Oncol. 2026 Jul 30;17:1271. doi: 10.1007/s12672-026-05692-9 (PMC13530083; doi:10.1007/s12672-026-05692-9)
Supplement: Supplementary file 1 [file 12672_2026_5692_MOESM1_ESM.docx]

**Supplementary Materials**

**Original blot-image statement.** The images below are the original, unprocessed, as-full-length-as-available image files supplied by the authors. No local adjustment of bands or background has been performed. Red boxes were added solely to indicate the regions used in the cropped panels of the main manuscript. Molecular-weight markers and unused image regions are retained where available.

**Lane-order statement.** For Supplementary Figures S2 and S3, the lane order from left to right after the molecular-weight marker is: HCT116 Control, HCT116 ATRi, HCT116 RT, HCT116 ATRi+RT, HCT116 KD Control, HCT116 KD ATRi, HCT116 KD RT, and HCT116 KD ATRi+RT. For Supplementary Figure S1, lanes are shown in the same order as in Figure 1A and represent HCT116 siNC and siP53 samples.

Supplementary Figure S1A. Original p53 blot corresponding to Figure 1A

The red box identifies the region used in the cropped p53 panel in Figure 1A. The molecular-weight marker is shown on the left.

Supplementary Figure S1B. Original GAPDH blot corresponding to Figure 1A

The red box identifies the region used in the cropped GAPDH panel in Figure 1A. The full available image canvas is retained.

Supplementary Figure S2A. Original total CHK1 blot corresponding to Figure 2A

The red box identifies the region used in Figure 2A. Lane order is provided in the lane-order statement above.

Supplementary Figure S2B. Original phospho-CHK1 (Ser345) blot corresponding to Figure 2A

The red box identifies the region used in Figure 2A. Lane order is provided in the lane-order statement above.

Supplementary Figure S2C. Original γH2AX (Ser139) blot corresponding to Figure 2A

The red box identifies the γH2AX band region used in Figure 2A. Lane order is provided in the lane-order statement above.

Supplementary Figure S2D. Original GAPDH blot corresponding to Figure 2A

The red box identifies the GAPDH band region used in Figure 2A. Lane order is provided in the lane-order statement above.

**Supplementary Figure S3A. Original phospho-Histone H3 (Ser10) blot corresponding to Figure 3C**

The red box identifies the pH3 band region used in Figure 3C. Lane order is provided in the lane-order statement above.

**Supplementary Figure S3B. Original cleaved caspase-3 blot corresponding to Figure 3C**

The red box identifies the cleaved caspase-3 band region used in Figure 3C. Lane order is provided in the lane-order statement above.

Supplementary Figure S3C. Original GAPDH blot corresponding to Figure 3C

The red box identifies the GAPDH band region used in Figure 3C. Lane order is provided in the lane-order statement above.

Supplementary Figure S4. Representative cell-cycle distributions 7 h after release from the second thymidine block

Representative cell-cycle profiles of HCT116 and HCT116 KD cells at the selected 7-h synchronization time point. The figure documents cell-cycle distribution at this time point and is not described as proof of maximal G2-phase enrichment.

**Consolidated figure legends**

**Supplementary Figure S1.** Original, unprocessed Western blot images corresponding to Figure 1A: p53 and GAPDH. Red boxes identify the regions used in the main figure.

**Supplementary Figure S2.** Original, unprocessed Western blot images corresponding to Figure 2A: total CHK1, phospho-CHK1 (Ser345), γH2AX (Ser139), and GAPDH. Red boxes identify the regions used in the main figure.

**Supplementary Figure S3.** Original, unprocessed Western blot images corresponding to Figure 3C: phospho-Histone H3 (Ser10), cleaved caspase-3, and GAPDH. Red boxes identify the regions used in the main figure.

**Supplementary Figure S4.** Representative cell-cycle distributions of HCT116 and HCT116 KD cells 7 h after release from the second thymidine block.
